# Climate-driven C_4_ plant distributions in China: divergence in C_4_ taxa

**DOI:** 10.1038/srep27977

**Published:** 2016-06-15

**Authors:** Renzhong Wang, Linna Ma

**Affiliations:** 1 State Key Laboratory of Vegetation and Environmental Change, Institute of Botany, the Chinese Academy of Sciences, No. 20 Nanxincun, Xiangshan, Beijing, 100093, China

## Abstract

There have been debates on the driving factors of C_4_ plant expansion, such as *P*CO_2_ decline in the late Micocene and warmer climate and precipitation at large-scale modern ecosystems. These disputes are mainly due to the lack of direct evidence and extensive data analysis. Here we use mass flora data to explore the driving factors of C_4_ distribution and divergent patterns for different C_4_ taxa at continental scale in China. The results display that it is mean annual climate variables driving C_4_ distribution at present-day vegetation. Mean annual temperature is the critical restriction of total C_4_ plants and the precipitation gradients seem to have much less impact. Grass and sedge C_4_ plants are largely restricted to mean annual temperature and precipitation respectively, while Chenopod C_4_ plants are strongly restricted by aridity in China. Separate regression analysis can succeed to detect divergences of climate distribution patterns of C_4_ taxa at global scale.

Modern ecosystems, such as tropical savannas, temperate grasslands and semi-deserts, have a significant component of C_4_ plants^1^. At global scale, only about 3% of total plant species is characterized by C_4_ photosynthetic pathway, C_4_ plants, however, account for roughly 25% of global terrestrial primary production, including important crops, weed plants and potential biofuels^2^,^3^. Understanding the occurrence and distribution of C_4_ biota can yield important information regarding to global primary productivity and to the effects of climate changes on ecosystem structures and functions^2^,^4^,^5^, as well as C_4_ plant’s past, present and future.

The abundance of C_4_ species in particular regions and their distribution in relation to climate have been well reported in North America^6^, Africa^7^,^8^, Europe^9^,^10^, Australia^11^, Middle East^12^,^13^, but has not been studied details in China and this knowledge is essential for formulating generalization regarding to global C_4_ occurrence and their relation with climate. The vast area and varied terrain in China with complex ecosystem components (*e.g*. rain forests, wet lands, temperate grasslands, deserts and tundra) and great climate changes contain more different C_4_ information. Moreover, the deserts in China and Asia differ markedly from the arid ecosystems of North America, Australia and Europe in the taxonomic groups of C_4_ species^5^,^14^. In China deserts and arid regions, Chenopodiaceae is the leading C_4_ family, but their distribution in relation to climate has not yet been addressed, this is very important for understanding the effects of climate changes on ecosystem structures and functions, particularly with the increasing of desertification in west China in recent decades^15^.

Although the occurrence and distribution of C_4_ plants have been documented at different scales over the past couple of decades, there have been debates on C_4_ plant expansion at large-scale^1^,^16^,^17^,^18^,^19^, for example, (i) what is the driving factor for C_4_ plant expansion, decrease in atmospheric CO_2_ concentration in the late Miocene or climate (both ancient and modern) variability^19^−^21^? It had been hypothesized that *P*CO_2_ decline caused C_4_ plant expansion rapidly during the late Micocene (~8 to 4 Ma)^1^,^16^,^17^, but some evidences suggested that C_4_ plant expansion was likely driven by addition factors, such as enhanced low-latitude aridity, seasonal precipitation^19^ and fire^20^ in the Miocene. The present-day global distribution of C_4_ grasses is largely restricted to warmer climate and precipitation, for strong positive relationships between C_4_ grass abundance and growing season temperature at continental scales^5^,^18^. Few found that the restriction of C_4_ grasses to warmer areas was due largely to their evolutionary history^2^. (ii) whether the different C_4_ taxa have similar climate distribution pattern in present-day at large-scale^21^,^22^? Indeed, there are few large data sets with which to examine occurrence and climate distribution pattern of different C_4_ taxa in modern vegetation at continental areas, resulting severely limits the accuracy understanding C_4_ plant expansion and the ecological implications.

## Results

**Figure 1:**
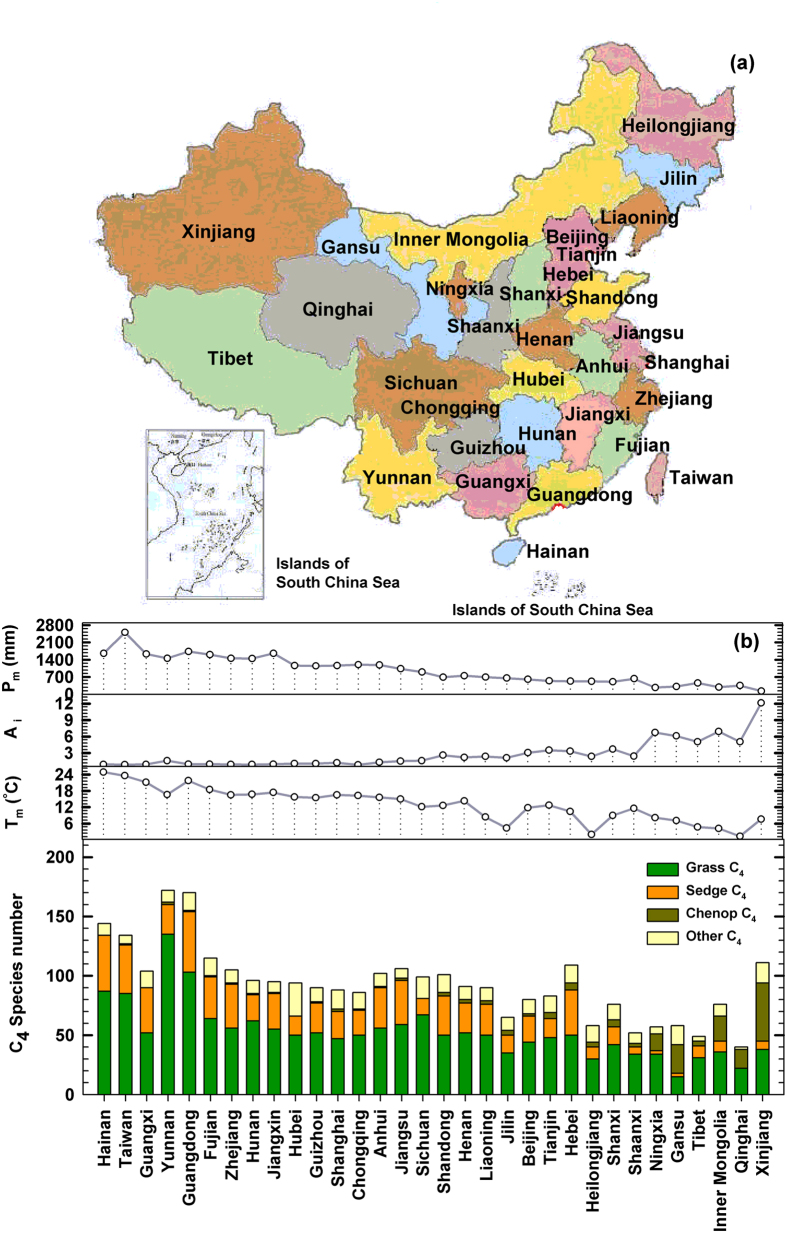
Geographical distribution (**a**), climate variables and numbers of C_4_ species occurrence in 32 provinces and municipalities of China (**b**). The map was generated by *ArcGIS 93 SLX* (http://www.esri.com/software/arcgis/). *Abbreviations* – P_m_, mean annual precipitation; Ai, arid index; T_m_, mean annual temperature; Chenop C_4_, C_4_ species in *Chenopodiaceae*. *Scientific Reports* remains neutral with regard to contested jurisdictional claims in published maps.

**Figure 2:**
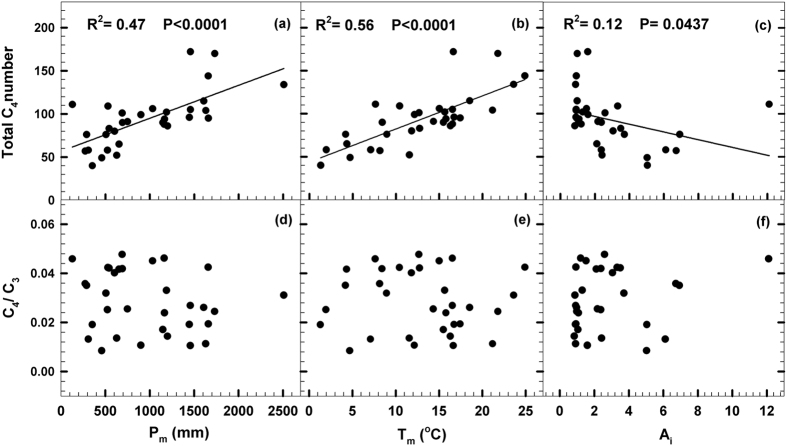
Regression of the total C_4_ species numbers and C_4_/C_3_ versus mean annual precipitation (P_m_), mean annual temperature (T_m_) and arid index (Ai) in China.

Of the total vascular plants (about 30000 species) in China, 371 species are identified with C_4_ photosynthesis in 11 families (Table 1; Supplementary Table S1), but 90.83% C_4_ species occurring in *Gramineae* (53.64%), *Cyperaceae* (19.67%) and *Chenopodiaceae* (17.52%; Chenopod, hereafter). Relative lower C_4_ plant occurrence is due largely to there is no tropical savannas (with more C_4_ grasses) in China. In general, total C_4_ species abundance decreases from south to north and from east to west in China (Fig. 1a,b). The total C_4_ species abundance in Heilongjiang (most northern territory) is only 1/3 of that in Yunnan (most southern territory), while that in western province of Qinghai is less than 1/3 of that in Taiwan (Fig. 1b). The total C_4_ species abundance is strongly and positively related with mean annual temperature (T_m_) (R^2^ = 0.56, P < 0.0001) and mean annual precipitation (P_m_) (R^2^ = 0.47, P < 0.0001; Fig. 2a–c). Multiple regression of the total C_4_ species abundance (Y_totalC4_) against climate variables shows that there is a strongly and positively correlation between Y_totalC4_ and T_m_, P_m_ and aridity (A_I_) as model:


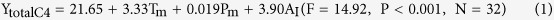


This indicates that these climate factors affect the distribution of total C_4_ species abundance in China. Stepwise multiple regression analysis exhibits that T_m_ has highest contributions (61.5%) to total C_4_ species distribution, while the impacts of P_m_ (2.0%) and A_I_ (2.6%) are relative less (Table 2).

**Figure 3:**
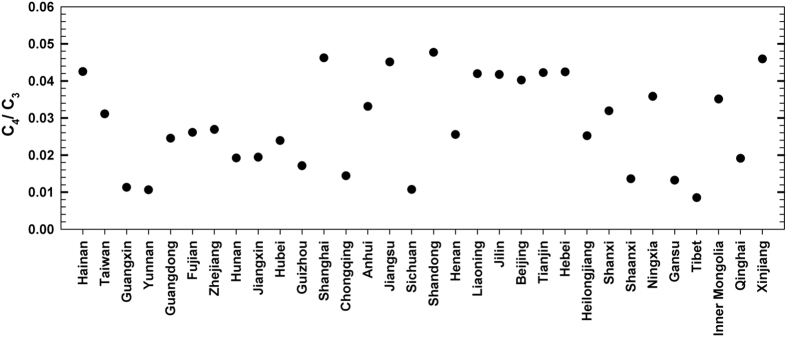
The C_4_/C_3_ fractions in 32 provinces and municipalities of China.

C_4_/C_3_ proportion in China flora is about 1.2%, ranging from 0.85% in Tibet to 4.77% in Shandong province (Fig. 3). Most humid southern provinces (*e.g*. Yunnan, Guangxi and Sichuan) have lower C_4_/C_3_, even though the occurrence of C_4_ species is high in these regions. There are no significant relations between C_4_/C_3_ proportions and climate variables in present-day vegetation in China (P > 0.05; Fig. 2d–f), indicating that C_4_/C_3_ proportion dose not exhibit certain ecological pattern, even the C_4_ occurrence dose significantly related with plant abundance at large-scale region.

**Figure 4:**
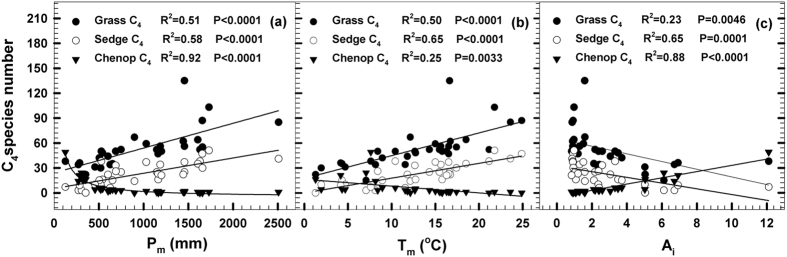
Relations of grass, sedge and Chenop C_4_ plant abundances with mean annual precipitation (P_m_), mean annual temperature (T_m_) and arid index (Ai) in China.

C_4_ distribution patterns predicted by total C_4_ species abundance appear to be insensitive to climate factors known to influence C_4_ occurrence and expansion because of the different adaptive strategies for C_4_ taxa to climate variables. Both grass and sedge C_4_ species abundances are strongly and positively related with P_m_ (R^2^ = 0.51, P < 0.001; R^2^ = 0.58, P < 0.001) and T_m_ (R^2^ = 0.50, P < 0.001; R^2^ = 0.65, P < 0.001), but significantly and negatively with aridity (R^2^ = 0.23, P < 0.01; R^2^ = 0.55, P < 0.001) (Fig. 4a–c). Multiple regressions of grass C_4_ species abundance (Y_grassC4_) and sedge C_4_ species abundance (Y_sedgeC4_) against climate variables manifest that Y_grassC4_ and Y_sedgeC4_ are strong correlated with T_m_, P_m_ and A_I_ as models:


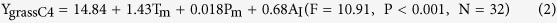



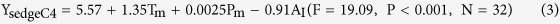


But stepwise multiple regression analysis demonstrates that grass C_4_ plant distribution is largely restricted to P_m_ (50.6%), and T_m_ and A_I_ functions are not significant (P > 0.05). Sedge C_4_ species is mainly limited to T_m_ (64.7%) and the impacts of P_m_ (0.2%) and A_I_ (2.3%) are very less and no significant (P > 0.05; Table 2).

On the contrary, Chenopod C_4_ plant abundance is strongly and positively related with A_I_ (R^2^ = 0.88, P < 0.001), and significantly and negatively with both P_m_ (R^2^ = 0.92, P < 0.001) and T_m_ (R^2^ = 0.25, P < 0.001; Fig. 4a–c). Stepwise regression of Chenopod C_4_ plant abundance against climate variables shows that there is a strong correlation between Y_Chenopod C4_ and T_m_, P_m_ and A_I_ as model:


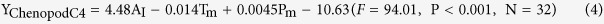


Stepwise multiple regression analysis exhibits that Chenopod C_4_ plants is confined to arid index (88.2%) in present-day vegetation at whole China. These suggest the distributions of C_4_ taxa are restricted to different climate factors at present-day vegetation in China.

## Discussion

There were many studies on the C_4_ plant expansion and distribution in relation to climate over the past couple of decades^1^,^16^,^17^,^18^,^19^, but only few manifested the detail floristic data of C_4_ occurrence in large regional scale (*e.g*. Europe^9^,^10^, Middle East^13^, Central Asia^14^). 371 identified C_4_ species within China account for roughly 20% of known C_4_ plants (about 1800 species global) and that is much greater than the percentage (~13%) of China vascular species to worldwide angiosperms, even though China is not a hot spot for C_4_ photosynthesis (Fig. 1; Supplementary Table S1). The total number of C_4_ species in China (Table 1), mainly grasses (53.64%), sedges (19.67%) and Chenopods (17.52%), is much greater than that in Europe^9^ and Middle East^13^. However, the number of Chenopod C_4_ species is only 1/3 of that in Middle East and 1.5 times of that in Mongolia^14^, probably because China arid regions is smaller than Middle East, but larger than Mongolia. This knowledge is essential for building global C_4_ plant database and formulating generalization regarding their relation to global climate.

What is the driving factor for C_4_ plant expansion and distribution remains controversial. The evidences of palaeovegetation and fossil tooth enamel indicated the global expansion of C_4_ plants may be related to lower *P*CO_2_ in the Miocene^1^,^16^,^17^, but some evidences suggested that C_4_ plant expansion was likely driven by climate variables and fire in both old world and present-day vegetation^19^,^20^. Our data clearly demonstrate that C_4_ plant distributions are restricted to mean annual climate variables (*e.g*. T_m_, P_m_ and A_I_) in the present-day vegetation in China (Fig. 2). From the south to the north, T_m_ governs the vegetation changes, while from the east to the west moisture gradient (P_m_) drives plant distributions^23^,^24^. Within China, the total C_4_ species abundance is strongly and positively related with T_m_ and P_m_, this suggests that there is remarkably strong tendency for C_4_ species to grow in hot and wet conditions, even though the stepwise multiple regression analysis exhibits that the impact of P_m_ is relative less (Table 2). This is much different with previous observations^6^,^11^,^25^, their evidences manifest that July average daily temperature is a critical factor for C_4_ distribution and the precipitation gradients seem to have much less impacts. Such significant difference is probably because almost 2/3 identified C_4_ species are perennial grasses, sedges and some Polygonaceae species, relative higher P_m_ and T_m_ are not only favor for their growth in growing seasons, but also for their survival in winters^15^,^23^,^24^. This is also partly supported by the observation of soil organic carbon and present vegetation which indicates that C_4_ fraction of Inner Mongolia grassland has increased by approximately 10% in the past decades because of increasing of temperature^26^.

The C_4_/C_3_ proportion in China flora is about 1.2% and much lower than 3% estimated at global scale^4^,^18^. This is mainly due to complex relief in China, 2/3 of the total area is mountains and plateaus^23^. More mountains and high moisture in southern provinces lead to vast forest vegetation with relative more tree species and lower fractions of grasses and Chenopod species, even the species abundances for both C_3_ and C_4_ plants are much high in the southern regions. In addition, lower C_4_ plant occurrence in China is for the absence of tropical savannas, which is estimated with more C_4_ grasses^1^, but large area of temperate grasslands and deserts (40% of China land) devotes considerable C_4_ plant resources^5^,^15^. There are no significant relations (P > 0.05) between C_4_/C_3_ proportions and climate variables in present-day vegetation in China (Fig. 2d–f), indicating that C_4_/C_3_ proportions do not show certain ecological pattern (Fig. 3), even though C_4_ occurrence dose significantly related with plant abundance at large-scale region.

Separate analysis for different C_4_ taxa succeed in detecting C_4_ distribution patterns accurately at continental scale, for the separate analysis can eliminate the noise signal from the C_4_ taxon with different responses to climate variables and adaptive strategies. Previous studies had found that grass and sedge C_4_ plants were largely restricted to July average daily temperature^6^,^11^,^18^,^25^. However, it is different in China, separate multiple regression analyses display that the grass and sedge C_4_ plant abundances are mainly restricted to P_m_ and T_m_ respectively (Table 2). Most grass C_4_ species are terrarium plants and their distributions are mainly restricted to P_m_, while almost all sedge C_4_ species are aquatic plants and the distributions of sedge C_4_ taxon is governed by T_m_ in China. This explanation is also supported by the relative higher proportions of perennial C_4_ grasses and sedges in the floristic data (Supplementary Table S1). Unlike the grass and sedge C_4_ plants, the distribution of Chenopod C_4_ taxon is drove by aridity (Table 2; Fig. 4). Chenopod C_4_ plants are favored arid regions with hot summer and sufficient summer precipitation, because most of Chenopod C_4_ plants are annual plant species, they can use seasonal precipitation efficiently in dry and hot conditions where the precipitation mainly falls in growing season, and these species can withstand severe droughts as seeds^15^. Even though there are a few studies on the occurrence of C_4_ Chenopods in particular regions^13^,^14^, but C_4_ Chenopod distribution in relation to large-scale climate change has remain largely unexplored and this is important for understanding the effects of climate changes on arid ecosystems with the increase of desertification in west China^15^. In the dry west China (*e.g.* Xinjiang, Qinghai and Inner Mongolia), 30–45% of the total C_4_ species is Chenopod plants, while that in the east and south China is less than 1%. Predominant C_4_ Chenopods in hot and arid ecosystems, as well as strong relations with aridity (Fig. 4), imply that the expansion of C_4_ Chenopods may be enhanced in China with an increase in hot and aridity worldwide as some climate- change scenarios suggested. Moreover, previous studies proved that the advantage of C_4_ plants in water-limited deserts are not considered critical for establishing C_4_ grass distribution pattern, and are commonly invoked to explain the dominance of C_4_ dicots^21^,^27^, even though they did not separate Chenopod C_4_ species from dicots.

Driving factor for C_4_ plant expansion at spatial and temporal large-scale is controversial. The palaeosol carbonate and fossil tooth enamel data implicated that the C_4_ plant expansion may have been due to decreasing of *P*CO_2_ in late Miocene^1^, but other evidences suggested that the development of low-latitude season aridity and changes in growing conditions led to the expansion of C_4_ plants at ~7 Ma^19^. These different explanations are mainly due to the lack of direct evidences and extensive data analysis. Our mass flora data analysis partly supports Pagain’s perspective^19^, but their evidences also can not explain the divergence distribution pattern of different C_4_ taxa. The divergence in C_4_ climate pattern implicates these C_4_ taxa may be with different area of origins, evolutionary histories^2^,^21^, expansion mechanism and adaptive strategies, because the Chenopod C_4_ taxon has a diametrically opposed distribution pattern with grass and sedge C_4_ taxa (Fig. 4). In the previous studies^6^,^18^,^25^, it had been found that grass and sedge C_4_ plants are governed by July average daily temperature, but the distribution of grass, sedge and Chenopod C_4_ species in China are largely restricted to P_m_, T_m_ and A_I_ respectively, for the mean annual climate variables (especially P_m_ and T_m_) can accurately describe the climate restrictions of plant distributions in China^23^,^24^. Edwards and Still also proved that the restriction of C_4_ grasses to warmer areas was due largely to their evolutionary history^2^.

Comparing with most previous researches^1^,^16^–19 this work provides detail floristic data of C_4_ occurrence in large regional scale based on China flora sources, which is essential for building worldwide C_4_ plant database, and also contributes direct evidence formulating generalization regarding the driving factors of C_4_ plant expansion. We suggest that the restriction of C_4_ distributions at continental scale is due to largely the annual climate variables (*e.g*. P_m_, T_m_ and A_I_) in present-day ecosystems in China. Different C_4_ taxa may exhibit diametrically opposite pattern in relation to climate at large-scale likely due to their differences in adaptations, area of origins and evolutionary histories^2^,^21^. Our findings suggest that the expansion of C_4_ Chenopods will increase with the increasing of aridity in western and central China as climate- change scenarios expected, on the contrary, that for grass and sedge C_4_ species may decrease in the future. This may have huge impacts on vegetation dynamics and primary plant production for the C_4_ plants accounts for roughly 1/4 of global terrestrial primary production^2^,^3^.

## Methods

### China topography and climate

China, occupied a large area, about 9.6 million km^2^ (3°51′–53°33.5′N; 73°33′–135°05′E), stretches 5,026 km across the East Asian landmass. It is primarily mountains, plateaus and plains country, 2/3 of the total area is mountains and plateaus^23^. Land elevation in the east plains is about 100–200 m above sea level (as l), while that in the southwest mountains and plateaus are as high as 4000–8000 m as l. The relief is very complicated with both latitudinal and longitudinal climate zones, mixed with steep altitudinal gradients in the northwest and southwest parts, leading great changes in climate.

The climate in China is extremely diverse due to its wide coverage, assortment of terrains and distances to the sea for different locations. Most of China lies in the temperate belt, with its south in subtropical belt and north in subarctic belt. In general, the average temperature in China is 11.8 °C, varying from 31 °C in July to −10 °C in January. Because of the Influences of both latitude and monsoon activities, temperatures vary a great deal, low temperature in winter is −40°C in Mohe, the northernmost of China, while in hot summer temperature can be as high as 50 °C in Turpan basin, Xinjiang. The average annual precipitation is about 620 mm, ranging from 150–400 mm in the western deserts and semi-deserts to 500–800 mm in the central region and vast flat plains, and 800–1000 mm in the eastern and coastal areas. The main nature vegetation types include tropical rain forest, wet land, grassland, desert and tundra^23^.

### Obtaining C_4_ taxon data and analysis

Local C_4_ taxon data of 32 provinces and municipalities (Fig. 1a) were collected from the C_4_ plant database of Plant Adaptation Strategy and Mechanism Group, Institute of Botany, CAS, Reipublicae Popularis Sinicae^23^, Catalogue of Life China and local flora sources^28^,^29^,^30^,^31^,^32^,^33^,^34^,^35^,^36^,^37^,^38^,^39^,^40^,^41^,^42^,^43^,^44^,^45^,^46^,^47^,^48^,^49^,^50^,^51^,^52^,^53^,^54^,^55^,^56^,^57^,^58^,^59^,^60^. Long-term (1950–2010) climate data were provided by the National Meteorological Information Center of China Meteorological Administration. C_4_/C_3_ proportion refers to the ratio of C_4_ species number to C_3_ species number in local flora. Regressions of C_4_ taxon (*e*.*g.* total C_4_ species abundance, grass and sedge C_4_ species abundances) against climate variables (*e.g.* temperature, precipitation and aridity) were performed using SPSS 17.0 in order to explain the distribution patterns of C_4_ taxa accurately at global scale. Stepwise multiple regression analyses between C_4_ taxon and climatic variables were used to quantify the critical restriction of C_4_ taxon. All statistical analyses were performed using SPSS 17.0 (SPSS for Windows, Chicago, IL, USA).

## Additional Information

**How to cite this article**: Wang, R. and Ma, L. Climate-driven C_4_ plant distributions in China: divergence in C_4_ taxa. *Sci. Rep.* **6**, 27977; doi: 10.1038/srep27977 (2016).

## Supplementary Material

Supplementary Information

## Figures and Tables

**Table 1:** The occurrence of C_4_ species in plant families and genera in China.

Family	Genera	Species number	% of total C_4_
*Dicotyledoneae*			
*Aizoaceae*	3	3	0.80
*Amaranthaceae*	3	18	4.85
*Chenopodiaceae*	17	65	17.52
*Crassulaceae*	1	1	0.27
*Euphorbiaceae*	1	2	0.54
*Nyetaginaceae*	1	2	0.54
*Polygonaceae*	1	5	1.35
*Portulaceae*	1	2	0.54
*Zygophyllaceae*	1	1	0.27
*Monocotyledoneae*			
*Cyperaceae*	12	73	19.67
*Gramineae*	72	199	53.64
Total	113	371	99.99

**Table 2:** Results of stepwise multiple regression analyses.

	P_m_		T_m_		A_i_	
	Partial R^2^	Probability	Partial R^2^	Probability	Partial R^2^	Probability
Total C_4_	0.020	0.243	0.615	0.000	0.026	0.180
Grass C_4_	0.506	0.000	0.031	0.176	0.002	0.710
Sedge C_4_	0.002	0.703	0.647	0.000	0.023	0.170
Chenop C_4_	0.027	0.006	0.000	0.945	0.882	0.000

Dependent variables: total C_4_, grass C_4_, sedge C_4_ and Chenop C_4_; Independent variables: mean annual precipitation (P_m_), mean annual temperature (T_m_) and arid index (A_i_). The values of parameter estimate refer positive/ negative relationships between the examined dependent variable and the independent variables.
