# Supplementary material for: Climate-driven C4 plant distributions in China: divergence in C4 taxa
Source: Sci Rep. 2016 Jun 15;6:27977. doi: 10.1038/srep27977 (PMC4908390; doi:10.1038/srep27977)
Supplement: Supplementary Information [file srep27977-s1.pdf]

Climate-driven C_4 plant distributions in China: divergence in C_4 taxa

Renzhong Wang, Linna Ma

State Key Laboratory of Vegetation and Environmental Change, Institute of Botany, the Chinese Academy of Sciences, No. 20 Nanxincun, Xiangshan, Beijing, 100093, China.

Correspondence and requests for materials should be addressed to R.W. (Email: wangrz@ibcas.ac.cn Tel: +86-10-62836550 Fax: +86-10-82595962*).*

Manuscript type: Research article

Number of tables: 2 Number of figures: 4

Supporting information: 1

Table S1. The occurrence of C_4 species in plant families and genera in China.

Family	Species
Dicotyledoneae	
Aizoaceae	*Gisekia pharnaceoides* L.
	Mollugo cerviana (L.) Ser.
	Trianthema portulacastrum L.
Amaranthaceae	*Alternanthera ficoides* (L.) R. Br.
	A. pungens H. B. K.
	A. repens (L.) R. Steud.
	Amaranthus albus L.
	A. blitoides S.Watson
	A. caudatus L.
	A. chlorostachys Willd.
	A. cruentus L.
	A. hybridus L.
	A. hypochondriacus L.
	A. lividus L.
	A. paniculatus L.
	A. retroflexus L.
	A. spinosus L.
	A. tricolor L.
	A. viridis L.
	Gomphrena celosioides Mart.
	G. globosa L.
Chenopodiaceae	*Aellenia glauca* (Bieb.) Aellen
	Anabasis aphylla L.
	A. brevifolia C. A. Mey.
	A. elatior (C. A. Mey.) Schischk.
	A. eriopoda (Schrenk) Benth. ex Volkens
	A. pelliotii Danguy
	A. phyllophora Kar. et Kir.
	A. salsa (C. A. Mey.) Benth.

A. truncata (Schrenk) Bunge

Atriplex belangeri Boiss.

A. cana C.A.Mey.

A. dimorphostegia Kar. et Kir.

A. glauca Pall.

A. laciniata L.

A. laevis C. A. Mey.

A. nummularia Lindl.

A. rosea L.

A. sibirica L.

A. tatarica L.

Bassia hyssopifolia (Pall.) O. Kuntze

Camphorosma lessingii Litv.

C. monspeliaca L.

Climscoptera affinis (C. A. Mey.) Botsch.

C. subcrassa (M.Pop.) Botsch.

Echinopsilon hyssopifolium (Pall.) Moq

Girgensohnia oppositiflora (Pall.) Fenzl

Halogeton glomeratus (Bieb.) C. A. Mey.

Haloxylon ammodendron (C. A. Mey.) Bunge

H. aphyllum (Minkw.) Iljin

H. persicum Bunge ex Boiss et Buhse.

Iljinia regelii (Bunge) Korov.

Kochia iranica Litv. ex Bornm

K. krylovii Litv.

K. laniflora (S. G. Gmel.) Borb.

K. melanoptera Bunge

K. prostrata (L.) Schrad.

K. scoparia (L.) Schrad.

K. scoparia (L.) Schrad. var. sieversiana (Pall.) Ulbr. ex Aschers et Graebn

K. sieversiana (Pall.) C. A. Mey.

Londesia eriantha Fisch et Mey

	Micropeplis arachnoides Bunge
	Petrosimonia litwinowii Korsh.
	P. sibirica (Pall.) Bunge
	Salsola arbuscula Pall.
	S. brachiata Pall.
	S. collina Pall.
	S. gemmascens subsp. *passerina* (Bunge) Botsch
	S. glauca Bieb.
	S. ikonnikovii Iljin
	S. lanata Pall.
	S. monoptera Bungr
	S. oppositiflora Pall.
	S. orientalia S. G. Gmel.
	S. passerina Bunge
	S. paulsenii Litv.
	S. pellucida Litv.
	S. praecox Litv.
	S. rigida Pall.
	S. rosacea L.
	S. soda L.
	Suaeda acuminata (C. A. Mey.) Moq.
	S. altissima (L.) Pall.
	S. dendroides (C. A. Mey.) Moq.
	S. heterophylla (Kar. et Kir.) Bunge
	S. monoica Forsk.
Crassulaceae	*Orostachys malacophyllus* (Pall.) Fisch.
Euphorbiaceae	*Euphorbia humifusa* Will.
	E. maculata L.
Nyetaginaceae	*Boerhaavia diffusa* L.
	B. erecta L.
Polygonaceae	*Calligonum ebi-nuricm* Ivanov ex Soskov
	C. gobicum (Bge. ex Meisn.) A. Los.

	C. Junceum (Fisch. et Mey.) Litv.
	C. mongolicum Turcz.
	C. pumilum A. Los.
Portulaceae	*Portulaca grandiflora* Hook.
	P. oleracea L.
Zygophyllaceae	*Tribulus terrestris* L.
Monocotyledoneae	
Cyperaceae	*Acorellus pannonicus* Palla
	Bulbostylis barbata (Rottb.) Kunth
	B. capillaris (Linn.) C. B. Clarke
	Cyperus amuricum Maxim.
	C. brevifolius (Rottb.) Hassk.
	C. compressus Linn.
	C. cyperinus Okwi
	C. cyperoides (Linn.) O. Ktze.
	C. digitatus Roxb.
	C. distans Linn. f.
	C. eragrostis Vahl
	C. esculentus L.
	C. exaltatus Retz.
	C. flabelliformis Rottb.
	C. globosus All.
	C. glomeratus Linn.
	C. imbricatus Retz.
	C. iria Linn.
	C. javanicus Houtt
	C. kyllingia Engl.
	C. malaccensis Lam.
	C. michelianus (Linn.) Link
	C. microiria Steud.
	C. nilagiricus Hochst. ex Steud.

C. nipponicus Franch. et. Savat.

C. nutans Vahl

C. orthostachyus Franch. et. Savat.

C. pilosus Vahl

C. polystachyus Rottb.

C. pygmaaeus Rottb.

C. rotundus Linn.

C. serotinus Rottb.

C. stoloniferus Retz.

C. tenuispica Steud.

Eleocharis japonica Miq.

E. palustris Bunge

E. tetraquetra Kom.

Fimbristylis aestivalis (Retz.) vahl

F. bisumbellata (Forsk.) Bubani

F. complanata (Retz.) Link

F. cymosa (Lam.) R. Br.

F. dichotoma (Linn.) Vahl

F. diphylloides Makino

F. dipsacea Kom.

F. ferruginea (Linn.) Vahl

F. globulosa (Retz.) Kunth

F. miliacea (Linn.) Vahl

F. monostachya (Linn.) Hassk.

F. nutans (Retz.) Vahl

F. ovate (Bum.f.) Kern.

F. pauciflora Chun et How

F. pierotii Miq.

F. polytrchoides (Retz.) Vahl

F. sericea (Poir.) R. Br.

F. sieboldii Miq.

F. squarrosa Vahl

	F. stauntoni Debeaux et Franch
	F. tetragona R. Br.
	Juncellus serotinus (Rottb.) C. B. Clarke
	Kyllinga brevifolia Diels
	K. monocephala Nees
	K. triceps Rottb.
	Lipocarpha chinensis (Osbeck) Tang et Wang
	L. microcephala (R. Br.) Kunth
	Mariscus compactus (Retz.) Druce
	M. cyperinus Vahl
	M. javanicus (Houtt.) Merr. et Metc.
	Pycreus polystachyus (Rottb.) P. Beauv.
	*P. sanguinolentu*s (Vahl) Nees
	P. sanguinolentus (Vahl) Nees
	P. unioloides (R. Br.) Urb.
	Rhynchospora rubra (Lour.) Makino
	Scirpus lateriflorus Gmel.
Gramineae	*Acrachne racemosa* Heyne et Roem. et Schult.) Ohwi
	Aeluropus lagopoides (L.) Trin. ex Thwaites
	A. littoralis Norlindh non Parl.
	A. micrantherus Tzvel.
	Alloteropsis cimicina (L.) Stapf
	A. semialata (R. Br.) Hitchc.
	Andropogon citratus DC.
	Apluda aristata Linn.
	A. mutica Linn.
	Aristida adscensionis L.
	Arthraxon hispidus (Thunb.) Makino
	Arundinella anomala Steud.
	A. hirta auctt. non (Thunb.) Tanak
	A. nepalensis Trin.
	Axonopus affinis A. Chase

A. compressus (Sw.) Beauv.

Bothriochloa glabra (Roxb.) A. Camus

B. intermedia (R. Br.) A. Camus

B. ischaemum (Linn.) Keng

B. pertusa (Linn.) A. Camus

Bouteloua curtipendula (Michx.) Torr.

B. gracilis (H. B. K.) Lag. ex Steud.

Brachiaria eruciformis (J. E. Smith) Griseb.

B. mutica (Forsk.) Stapf

B. ramosa (L.) Stapf

B. semiundulata (Hochst.) Stapf

B. subquadripara (Trin.) Hitchc.

Buchloe dactyloides (Nutt.) Engelm.

Capillipedium parviflorum (R. Br.) Stapf var. spicigerum (Benth.) Hsu

Cenchrus calyculatus Gavan.

C. echinatus L.

C. setigerus Vahl

Chloris barbata Sw.

C. cruciata (L.) Sw.

C. gayana Kunth

C. inflata Link.

C. petraea Sw.

C. virgata Sw.

Chrysopogon gryllus (Linn.) Trin.

Cleistogenes caespitosa Keng

C. chinensis (Maxim.) Keng

C. foliosa Keng

C. kitagawai Honda

C. songorica (Roshev.) Ohwi

C. sguarrosa (Trin.) Keng

Coix aquatica Roxb.

C. lacryma-jobi Linn.

Crypsis aculeata (L.) Ait

C. schoenoides (L.) Lam.

Cymbopogon citratus (DC.) Stapf

C. flexuosus (Nees ex Steud.) Wats.

C. jwarancusa (Jones) Schult.

C. martinii (Roxb.) Wats.

C. nardus (Linn.) Rendle

C. tortilis (Presl) A. Camus

C. winterianus Jowitt

Cynodon arcuatus J. S. Presl ex Presl

C. dactylon (L.) Pers.

Dactylocatenium aegyptiacum Willd.

Desmostachya bipinnata (L.) Stapf

Dichanthium annulatum (Forssk.) Stapf

D. aristatum (Poir) C. E. Hubb.

Digitaria adscendens (H. B. K.) Henr.

D. bicornis (Lam.) Roem. et Schult.

D. ciliaris (Retz.) Koel.

D. ischaemum (Schreb.) Schreb.

D. sanguinalis (L.) Scop.

Dinebra retroflexa (Vahl) Panz.

Echinochloa colonum (L.) Link

E. crusgalli (L.) Beauv.

E. frumentacea Roshev. non Link

E. utilis Ohwi et Yabuno

Eleusine coracana (L.) Gaertn.

E. indica (L.) Gaertn

Enneapogon borealis (Griseb.) Honda

Eragrostis amabilis (L.) Wight et Arn. ex Hook. et Arn.

E. chariis Merr. mon Hitchc.

E. cilianensis (All.) Link ex Vignclo-Lutati

E. ciliaris Merr. non Link

E. curvula (Schrad.) Nees

E. ferruginea (Thunb.) Beauv.

E. minor Host

E. pilosa (L.) Beauv.

E. poaceoides Beauv.

E. unioloides (Retz.) Nees ex Steud

Eremochloa bimaculata Hack.

E. ophiuroides (Munro) Hack.

*E. foveolatu*s (Del.) Stapf

Erianthus fastigiatus Henry non Nees

E. giganteus (Walt.) Muhl.

E. ravennae (Linn.) Beauv.

Garnotia patula (Munro) Benth. var. strictor Santos

G. triseta Hitchc.

Hackelochloa granularis (Linn.) Kuntze

Hemarthria sibirica (Gand.) Ohwi.

Heteropogon contortus (Linn.) P. Beauv. ex Roem.

Hyparrhenia filipendula (Hochst.) Stapf

H. rufa (Nees) Stapf

Imperata arundinacea Cyrillo

I. cylindrica (Linn.) Beauv.

Isachne dispar Trin.

Leptoloma cognatum (Schult.) A. Chase

Manisuris altissima (Poir.) Hitchc.

Melinis minutiflora Beauv.

Miscanthus sacchariflorus (Maxim.) Benth.

M. sinensis Anderss

Muhlenbergia schreberi J. F. Gmel.

M. racemosa (Hack.) Makino.

Ophiuros exaltatus (Linn.) Kuntze

Panicum ambiguum Trin.

P. coloratum F. Muell non L.

P. decompasiium Rendle

P. dichotomiflorum Michx.

P. geminatum Honda non Forssk

P. indicum L. non Mill.

P. maximum Jacq.

P. miliaceum L.

P. polygamum Sw.

P. purpurascens Raddi ex Opiz

P. repens L.

P. reptans L.

P. virgatum L.

Paspalum ciliatifolium Trin.

P. commersonii Lam.

P. conjugatum Berg.

P. dilatatum Poir.

P. distichum Rendle non L.

P. notatum Flugge

P. orbiculare Forst.

P. paspaloides (Michx.) Scribn.

P. scrobiculatum L.

P. urvillei Steud.

P. vaginatum Sw.

Pennisetum alopecuroides (L.) Spreng.

P. centrasiaticum Tzvel.

P. cladestinum Hochst. ex Chiov.

P. flaccidum Rendle non Griseb.

P. glaucum (L.) R. Br.

P. orientale Rich.

P. purpureum Schum.

P. setosum (Swartz) Rich.

P. typhoideum L. Rich

Perotis hordeiformis Nees ex Hook. et Arn.

P. indica (L.) Kuntze

Pogonatherum paniceum (Lam.) Hack.

Polytrias amaura (Buse) Kuntze

Pseudopogonatherum irritans (R. Br.) A. Camus

Rhynchelytrum repens (Willd.) Hubb.

R. roseum (Nees) Stapf et Hubb. ex Bews

Rottboellia exaltata Linn. f.

Saccharum arundinaceum Retz.

S. officinarum L.

S. sinense Roxb.

S. spontaneum L.

Sehima nervosa (Rottl.) Stapf

Setaria faberii Herrm.

S. geniculata (Lam.) Beauv.

S. glauca (L.) Beauv.

S. italica (L.) Beauv.

S. lutescens (Weig.) F. T. Hubb.

S. pallidifusca (Schumach.) Stapf et Hubb.

S. palmifolia (Koen.) Stapf

S. pumila (Poir.) Roem. et Schult.

S. verticillata (L.) Beauv.

S. viridis (L.) Beauv.

Sorghum bicolor (L.) Moench

S. caffrorun (Retz.) Beauv.

S. dochna (Forssk.) Snowden

S. halepense (Linn.) Pers.

S. japonicum Roshev.

S. propinquum (Kunth) Hitchc.

S. sudanense (Piper) Stapf

S. technicum (Koern.) Roshev.

S. vulgare Pers.

Spartina alterniflora Lois.

	S. maritima (Curt.) Fenard.
	S. patens (Ait.) Muhl.
	S. townsendii H. et J. Goroves
	Spodiopogon sibiricum Trin.
	Sporobolus diander (Retz.) Beauv.
	S. elongatus Hichc. non R. Br.
	S. indicus (L.) R. Br.
	S. littoralis (Lam.) Kunth
	S. poiretii (Roem. et Schult.) Hitchc.
	Stenotaphrum subulatum Trin.
	Themeda triandra Forssk.
	Thuarea involuta (Forst.) R. Br. ex Roem. et Schult.
	Tragus bertesonianus Schult.
	T. biflorus sensu Schult.
	T. mongolorum Ohwi
	Tripogon chinensis (Franch.) Hack.
	T. purpurascens Duthie
	Tripsacum dactyloides (Linn.) Linn.
	Urochloa panicoides Beauv.
	Vetiveria zizanioides (Linn.) Nash
	Zea mays Linn.
	Zoysia japonica Steud.
	Z. matrella (L.) Merr.
	Z. tenuifolia Willd. ex Trin.
Total	371
